# False memory formation in cannabis users: a field study

**DOI:** 10.1007/s00213-019-05309-w

**Published:** 2019-06-28

**Authors:** Lilian Kloft, Henry Otgaar, Arjan Blokland, Alicja Garbaciak, Lauren A. Monds, Johannes G. Ramaekers

**Affiliations:** 1 Faculty of Psychology and Neuroscience, Maastricht University, Maastricht, The Netherlands; 2 Faculty of Law, Catholic University of Leuven, Leuven, Belgium; 3 Faculty of Medicine and Health, The University of Sydney, Sydney, Australia

**Keywords:** Cannabis, THC, Recognition memory, False memory, DRM paradigm

## Abstract

**Rationale:**

Cannabis use is widespread and has previously been associated with memory impairments. However, the role of cannabis in relation to false memory production, i.e., memories of events that were not experienced, is less well-understood.

**Objective:**

The aim of the current field study was to examine the impact of cannabis use on false memory production.

**Methods:**

The Deese/Roediger-McDermott (DRM) paradigm was used to induce false memories. In this paradigm, participants study word lists that are associatively related to a non-presented word, termed the *critical lure*. In a later memory test, true recognition rates and false alarm rates toward critical lures and unrelated items are assessed. Memory performance was compared between three groups: regular cannabis consumers who were acutely intoxicated (*n* = 53), regular cannabis consumers who were sober (*n* = 50), and cannabis-naïve controls (*n* = 53). The participants were approached in Dutch coffee shops (cannabis outlets) and cafes and asked to participate in our study. After collecting general information on their cannabis use, they were subjected to the DRM procedure.

**Results:**

Although false memory rates for critical lures did not statistically differ between groups, both intoxicated and sober cannabis consumers falsely recognized more unrelated items than control participants. Also, individuals without a history of cannabis use demonstrated higher memory accuracy compared with the intoxicated group.

**Conclusion:**

It is concluded that cannabis intoxication and history of cannabis use induce a liberal response criterion for newly presented words for which the level of association with previously learned words is low and uncertainty is high.

## Introduction

Cannabis is the world’s most widely used “illicit” drug, with an estimated global prevalence of 2.7–4.9% and a lifetime use of 78% (United Nation Office on Drugs and Crime 2017; Winstock et al. 2017). Given the recent legalization of recreational (e.g., Canada; Denny and Shumaker 2018) and medical (e.g., Australia; Thomsen 2016) uses of cannabis in several countries, as well as advances in technologies to deliver cannabis in less harmful ways, the way is being paved for increased cannabis consumption in the Western world. In comparison with alcohol and tobacco, cannabis has been ranked low on physical harm, dependency, and social harm (van Amsterdam et al. 2015). Although cannabis appears to have potential for medical use such as pain relief (Urits et al. 2019), cannabis may also induce cognitive impairment, particularly in the domain of memory (Broyd et al. 2016). A lack of knowledge exists, however, about the impact of cannabis on the formation of false memories (remembrance of events/details that were not experienced; Loftus 2004; Otgaar et al. 2016).

Cannabis use has been associated with memory impairments both during acute intoxication (Ranganathan and D’Souza 2006) and during abstinence in long-term users (Solowij and Battisti 2008). According to the majority of the research on this topic, cannabis use appears to primarily impair memory in the domains of verbal learning and declarative memory (Broyd et al. 2016; Schoeler and Bhattacharyya 2013; Theunissen et al. 2014). That is, the primary active cannabinoid tetrahydrocannabinol (THC) acutely elicits reliable, dose-dependent impairments in immediate and delayed verbal memory performance, most often measured using word list learning tasks testing both recall and recognition memory (although recognition memory is less consistently affected, e.g., Broyd et al. 2016; Hart et al. 2010).

Specifically, research has shown that acutely intoxicated participants recall fewer studied items compared with sober participants, a deficit which cannot be accounted for by cannabis-induced disruption of attentional processes (Ranganathan and D’Souza 2006). Such memory impairment has been found to persist in the unintoxicated state in long-term users, although some studies have also reported an improvement or recovery of memory functioning after a period of abstinence (see Broyd et al. 2016). However, interpretation of findings is often complicated by confounding factors such as frequency and duration of use, and differences across studies in terms of route of administration, sample size, and variations in cannabis strains in terms of the percentage of THC and cannabidiol (CBD) content and dosage (e.g., Ranganathan and D’Souza 2006).

While declarative memory impairments are a well-known consequence of cannabis use, an under-investigated avenue is the possibility of cannabis impacting false memory formation (memories for non-experienced events). A robust and reliable way to experimentally create false memories is by using the Deese/Roediger-McDermott (DRM) paradigm, in which lists of associatively related words are presented during encoding (e.g., *bed*, *dream*, *wake*, *rest*, *tired*) in which one highly related theme word (the “critical lure”: *sleep*) is not presented (Deese 1959; Roediger and McDermott 1995). Research shows that critical lures are often incorrectly recalled or recognized as having been presented before, thereby forming false memories, and acceptance rates for critical lures are often as high as acceptance rates for presented items (Brainerd et al. 2008; Reyna and Lloyd 1997).

Previous research on false memories and cannabis using the DRM paradigm is limited and has produced mixed results. Ballard et al. (2012) investigated the effects of THC (0-, 7.5-, and 15-mg capsules) at encoding on DRM true and false recognition performance 48 h later and found that THC impaired true recognition memory at both doses. False memory was not affected compared to placebo but was reduced compared to a memory-enhancing drug (dextroamphetamine; AMP) condition. Moreover, drug effects on true and false memory were positively correlated. In contrast, in a more recent study from the same lab (Doss et al. 2018), encoding took place under sober conditions while retrieval 48 h later occurred during intoxication. They found that selectively administering THC (15-mg capsule) during memory retrieval increased false recollection. Together these studies indicate that THC might affect encoding and retrieval differentially.

The relationship between cannabis and false memories was further investigated by Riba et al. (2015) in abstinent cannabis users. Daily cannabis consumers (*n =* 16), who were abstinent for 4 weeks, were compared with a matched cannabis-naïve control group. While no statistically significant difference between groups in true memory performance was found, abstinent cannabis users showed an increased susceptibility to false memory formation. However, these studies have limitations in that they do not provide any information on the acute effects of individually determined doses of smoked THC on false memory production.

Several studies investigating the acute effects of THC on word list tasks other than the DRM paradigm have reported an increase in intrusions (recalling non-presented items, e.g., Miller and Cornett 1978; Miller et al. 1977; Pfefferbaum et al. 1977) and false alarms (recognizing non-presented items, e.g., Hart et al. 2010; Ilan et al. 2004) in frequent and non-frequent users. This indicates that intoxicated individuals might display an increased tendency to recall items that were never presented to them.

Investigating the possibility of cannabis use leading to false memories may be of particular relevance in legal or forensic contexts. Individuals who use cannabis may be involved in legal cases as witnesses or suspects where they have to provide accurate accounts of events. To date, two studies examining the acute effects of cannabis on eyewitness memory (e.g., memory for a crime film or staged crime) have been conducted (Vredeveldt et al. 2018; Yuille et al. 1998). These studies did not find that cannabis intoxication led to a higher rate of incorrect recall. While both studies assessed the impact of cannabis on true memory recall, including a measure of incorrect details, neither study assessed the sensitivity of cannabis users for false memory production. A study that directly manipulates false memory such as through the use of the DRM paradigm can better address the question whether cannabis use heightens the susceptibility to false memory.

The present study was designed to assess the impact of cannabis intoxication or recent use on false memory production with a classical DRM paradigm in a real-life field setting. Dutch regulations permit the presence of cannabis outlets (“coffee shops”), alcohol-free establishments in which adults (18+) under certain conditions can buy and consume cannabis, creating a unique setting for the investigation of cannabis use (Niesink et al. 2015). The present study included three groups: regular cannabis users under acute influence, sober regular cannabis users, and sober controls with limited lifetime cannabis exposure. Memory performance of these groups was compared in order to assess immediate and residual effects of cannabis use, relative to controls. Based on previous findings (e.g., Riba et al. 2015), we predicted false memory performance to differ between all the three groups. Specifically, acutely intoxicated users were expected to exhibit highest rates of false recognition compared with the other two groups, but given potential residual effects, sober users were also expected to show impairment. It was anticipated that the control group would exhibit low rates of false recognition.

## Method

### Participants

An a priori power analysis was conducted using G*Power (Faul et al. 2007), with an expected medium effect size (Cohen’s *d* = 0.5), a power level of 80%, and an alpha of 0.05, resulting in a required total sample size of *N* = 159. The present sample (*N =* 159) included 53 cannabis users under acute influence of cannabis (46 males, 6 females), 53 sober but regular cannabis users (45 males, 8 females), and 53 controls (20 males, 33 females). After reviewing the data, three participants of the sober group were excluded from the analyses due to exceeding the age limit (*n* = 1) or indicating that they had in fact consumed cannabis (*n* = 2), resulting in *n* = 50 for this group. Common inclusion criteria for all groups were as follows: age between 18 and 30 years old, being comfortable with taking the study in English, and no alcohol consumption on the day of the experiment. Specific inclusion criteria for the cannabis intoxication group were as follows: acute intoxication (having smoked cannabis no longer than 60 min prior to memory testing), orientation in space and time (naming the day and place and solving a simple math problem), and regular use of cannabis (at least 1/month). Specific inclusion criteria for the cannabis sober group were as follows: no use of cannabis on the day of the memory test, and regular use of cannabis. The inclusion criteria for the control group were no use of cannabis in the past 24 h and a lifetime cannabis use of ≤ 10 occasions. A detailed summary of the demographics is given in Table 1.

**Table 1:** Subject characteristics for all groups

Demographic variables	Cannabis intoxication group (*N* = 53)	Cannabis sober group (*N* = 50)	Control group (*N* = 53)	*p*
Age in years (mean, SD)	21.6 (2.5)	21.1 (3.1)	22.5 (2.8)	0.06^1^
Sex (#)				
Male	46	43	20	< 0.001^2^
Female	7	7	33	
Native language (#)				0.26^3^
Dutch	26	30	30	
English	4	5	0	
Other language	17	16	12	
Missing data	6	0	11	
Level of education^a^ (#)				0.06^3^
No degree	1	4	0	
High school	34	33	33	
Bachelor’s degree	13	10	14	
Master’s degree	1	1	6	
Other^b^	4	3	0	
Lifetime diagnosis of a psychiatric disorder (#)				0.06^3^
Never diagnosed	52	45	47	
ADHD/ADD	0	2	1	
Mood disorder	1	3	4	
Anxiety disorder	0	0	1	
PTSD	0	1	0	
Autism	0	1	0	

^a^Level of education was measured in terms of highest level of education completed

^b^Other refers to higher professional education (Dutch: HBO), secondary vocational education (Dutch: MBO), or not specified (missing)

^1^Based on ANOVA

^2^Based on Pearson’s chi-square test

^3^Based on Fisher’s exact test

The cannabis intoxication group contained regular cannabis users recruited at one of several coffee shops in the city of Maastricht. Participants in this group rated their subjective high (feeling of intoxication) on a 100-mm visual analogue scale to be 5.9 (*SD* = 2.4) on average. Moreover, the group reported having smoked an average of 0.7 g (*SD* = 0.7) of cannabis that day, with 0.4 g (*SD* = 0.3) being in the last hour before testing. The majority had consumed cannabis through smoking a joint, i.e., a cannabis cigarette (98%). Regarding the type of cannabis used, 47% had consumed a hybrid strain, 26% had used a sativa strain, 19% had used an indica strain, and 6% had used hashish (2% missing values; data based on classification displayed in coffee shop). Based on the type of cannabis strain that participants in this group indicated using, several online cannabis strain databases (such as www.wikileaf.com/strains). The estimated average THC percentage was 19.1% (*SD* = 8.4, 19 missing values).

The cannabis sober group included sober coffee shop attendees, who were also regular users but who reported not having used any cannabis that day. The majority reported having used cannabis the last time on the day before (72.5%). The summary of cannabis use history for both the cannabis intoxication and the cannabis sober group is provided in Table 2. Furthermore, the control group consisted of non-users recruited at cafes in Maastricht or in the main buildings of the Faculty of Psychology and Neuroscience of Maastricht University (e.g., in the cafeteria or common area outside the library).

**Table 2:** History and patterns of cannabis use for both groups of cannabis users, mean (SD)

	Cannabis intoxication group (*N* = 53)	Cannabis sober group (*N* = 50)	*p*
Age of first use	15.5 (2.0)	15.3 (1.9)	0.67^1^
Age of regular use	17.9 (2.0)	17.5 (2.2)	0.28^1^
Frequency/week	6.3 (1.2)	5.4 (2.0)	< 0.01^1^
Frequency/month	26.8 (5.7)	22.9 (9.0)	< 0.01^1^
Grams used/week	5.9 (3.3)	7.3 (9.0)	0.29^1^
Grams used/month	25.5 (14.8)	30.6 (38.9)	0.39^1^
Average amount of cannabis per joint (g)	0.3 (0.1)	0.3 (0.1)	0.77^1^
Percentage cannabis per joint	53.5 (19.8)	50.9 (18.6)	0.51^1^
Percentage self-rated light versus heavy user	30/70	28/72	0.81^2^

^1^Based on independent samples *t* test

^2^Based on Pearson’s chi-square test

Across groups, fourteen participants (9.0%) indicated to have been diagnosed with a psychiatric disorder and a total of five participants indicated being under acute influence of medication at the moment of testing (Ritalin (1), antidepressants (2), antipsychotics (1), antihistamines (1)). These participants were retained in the sample in order to keep the sample diverse and representative of the population of individuals who use cannabis. However, their potential for confounding was evaluated by conducting the analyses both including and excluding these cases (see “Statistical analysis”).

Ethical approval for this study was obtained from the Ethical Review Committee Psychology and Neuroscience (ERCPN) from Maastricht University. All data and materials can be found on the Open Science Framework: https://osf.io/h3gsz/.

### Materials

#### The Deese/Roediger-McDermott paradigm

True and false memories were measured with the DRM paradigm, which has been shown to robustly elicit spontaneous false memories in previous research (Gallo 2010). The task consists of two phases: a study phase, in which participants study the stimuli; and a testing phase, in which a recognition memory is administered. In each phase, the word lists were administered to the participant as auditory stimuli. A separate recording was made for each phase. Stimuli were spoken and recorded by a male native Canadian English speaker at a rate of 1 word every ~ 2 s for the study phase and a rate of 1 word every ~ 6 s for the testing phase. The stimuli were administered to participants via over-ear headphones (Sony, Model MDR-ZX110). The instructions were repeated before each phase.

In the study phase, 10 DRM word lists were presented, each containing 10 words (lists: bread, cold, doctor, fruit, man, girl, sleep, soft, sweet, thief; adopted from Stadler et al. 1999). Normative data have shown that these lists vary in both their backward associative strength (BAS, index of the associative strength between the list items and the critical item) and their inter-item associative strength, with mean BAS ranging from 0.39 (sleep) to 0.06 (man), and false recognition of the critical lure ranging from 84% (cold) to 45% (fruit; see Roediger et al. 2001; Stadler et al. 1999). The recognition phase included the auditory presentation of 60 words, consisting of 30 previously presented words (words 1, 3, and 5 from each list), 10 new words semantically related to the lists from the study phase (critical lures), and 20 new unrelated words (unrelated items, adopted from other, non-presented DRM lists). The participants were instructed to make a *yes* (studied) or *no* (unstudied) judgment for the 60 words on a score sheet containing 60 yes/no columns. The item number was repeated before each word to prevent errors in completing the score sheet. In between the phases, the demographics and cannabis use questionnaire and a filler task (coloring a mandala) were administered (total time 10 min). The stimuli from both phases including their BAS are displayed in Appendix Tables 3 and 4.

Outcome measures included hit rate (the proportion of studied words correctly recognized at test), false alarm rate for critical lures (the proportion of critical lures, i.e., new, strongly related words, that are incorrectly recognized at test, a measure of false memory), false alarm rate for unrelated items (proportion of incorrect recognition of new, unrelated words), and net accuracy (total hits divided by all yes responses, an indication of overall ability to discriminate between studied and unstudied items).

#### Demographics and cannabis use questionnaire

For all groups, the questionnaire contained five self-report screening questions to assess if the respective group inclusion criteria were met, and five items on sociodemographic variables (sex, native language, highest completed level of education, lifetime diagnosis of a psychiatric disorder, current use of medication). The questionnaire administered to both groups of cannabis users contained additional items on their patterns of cannabis use (age of first use, age of regular use, tendency to use cannabis/hash/both, usual method of consumption, frequency of use per week/month, grams used per week/month, grams per joint, percentage cannabis/tobacco per joint, and a binary rating whether they consider themselves a heavy vs. light user). For the experimental group, the questionnaire included further items regarding their acute cannabis use (grams used today and within last hour, cannabis strain used, method of consumption). These subjects also rated their subjective high on visual analogue scales (100 mm, ranging from “totally not under the influence of cannabis” to “very much under the influence of cannabis”).

### Procedure

Data were collected between May 2017 and October 2018 in several centrally located coffee shops in Maastricht. Testing took place during daytime only (between 12 and 6 PM). The control group was recruited from local cafes and local university buildings. Owners or employees of these establishments were approached for their consent to recruit and test participants there. Potential participants were approached inside the establishments and made aware of the study by the experimenter. Specifically, potential participants for the cannabis intoxication group were approached in a coffee shop after they had consumed a cannabis product. For the cannabis sober group, participants were recruited in a coffee shop by approaching them directly after they had bought a cannabis product and sat down at a table. For the control group, anyone sitting in the cafe or Maastricht University common areas who appeared to be between the ages of 18 and 30 was approached. Before the experiment, potential participants were verbally screened for the inclusion criteria. Concerning the cannabis intoxication group, three additional screening questions were asked to ensure that these participants were oriented in time and space (naming the day, place, and solving a simple math problem). In line with good practice recommendations in intoxication research (Aldridge and Charles 2008), this was done in order to screen out severely intoxicated individuals, given concerns regarding their capacity to give informed consent. Eligible participants were informed that it was a study on memory in cannabis users and signed the informed consent form.

The DRM assessments were then conducted in the area where the participant was seated. Participants were handed over-ear headphones, and it was made sure they were comfortable with the volume and knew how to adjust it. Participants listened to the study phase recording (~ 5 min). Next, the demographics and cannabis use questionnaire and the filler task were administered (timed to take 10 min in total). Participants then listened to the testing phase recording, entering their recognition responses on the score sheet (~ 7 min). Upon completion, the participants were debriefed and rewarded for their participation with either a candy or a monetary remuneration (voucher worth €5–10).

### Statistical analysis

First, equivalence of all groups was tested by performing comparative analyses on key demographic variables. Group differences were tested for the following variables: age (analysis of variance), sex (Pearson’s chi-square), native language, level of education, and diagnosis of a psychiatric disorder (Fisher’s exact test for latter three). Moreover, intoxicated and sober cannabis users were compared with regard to variables of cannabis use history, using independent samples *t* tests for continuous and Pearson’s chi-square for categorical variables. Variables found to statistically significantly differ between groups were entered as covariates in general linear model analyses. As part of the exploratory analyses, findings of the covariate analyses were compared with the findings from the first-level analyses.

A first-level analysis of variance (ANOVA) was conducted on all four DRM outcome measures with group as a between-subjects factor (3 levels). When a significant overall group difference was detected, pairwise comparisons were conducted using the Bonferroni post hoc test. A difference was considered significant for *p* values < 0.05. Cohen’s *d* was calculated as an effect size estimate. These analyses were repeated, this time excluding those participants who had reported a lifetime diagnosis of a psychiatric disorder or use of medication (*n* = 16). Removal of these participants did not change the outcome; therefore, these analyses are not reported. Finally, secondary analyses were conducted to explore factors that may have contributed to the outcome, such as cannabis use history (e.g., frequency, onset of regular use, user type). Here, independent samples *t* tests were conducted to compare differences in cannabis use between light and heavy cannabis users and to examine group differences on the DRM outcome measures.

The assumptions underlying all analyses were checked. For independent samples *t* tests, this was done by examining Levene’s test for equality of variances. If this test was significant, then the more robust Welch *t* test was conducted in place of the regular independent samples *t* test, with corrected degrees of freedom reported to two decimal places. For ANOVA, assumptions were checked by visual inspections of Q-Q plots for normality and by examination of Levene’s test for equality of variances. No gross violations of assumptions were detected for ANOVA.

## Results

### Group characteristics

The sociodemographic characteristics of the sample are displayed in Table 1. Analyses of these characteristics revealed that the three groups did not statistically significantly differ in age, level of education, native language, and diagnosis of a psychiatric disorder. However, groups differed statistically significantly with regard to sex distribution. This variable was entered as a covariate in a multivariate general linear model analysis with all four DRM parameters as dependent variables (DVs) and group as a fixed factor. Sex was found to be statistically significantly associated with one of the DRM parameters (false alarms for critical lures); thus, this factor was further investigated in an exploratory analysis (see below). Cannabis use characteristics (see Table 2) generally did not differ between cannabis using groups. Weekly and monthly frequency use differed significantly between the groups, but was very minor.

### Hit rates

**Fig. 1:**
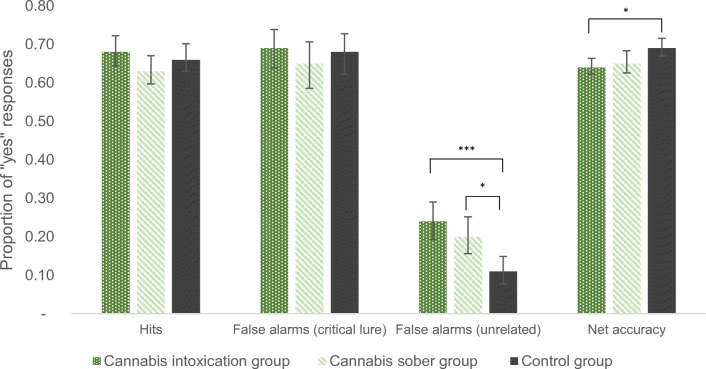
DRM memory performance parameters with 95% confidence intervals as error bars (rates in proportions, *p < 0.05, ***p < 0.001)

All mean scores for the DRM analyses can be inspected in Fig. 1. A one-way analysis of variance (ANOVA) was conducted on the proportion of hits. The hit rates did not statistically differ between the three groups (*F*(2, 153) = 1.75, *p* = 0.18, partial eta squared = 0.02).

### False alarm rates

A one-way ANOVA was also conducted on the proportion of false alarms (incorrect recognition of new words). When focusing on critical lures, cannabis use did not exert any notable effect on the formation of false memories for critical lures (*F*(2, 153) = 0.64, *p* = 0.53, partial eta squared = 0.01). However, when looking at false alarms in response to unrelated items, a statistically significant difference was detected between groups and was associated with a medium-large effect size (*F*(2, 154) = 8.99, *p* = 0.0002, partial eta squared = 0.11). The Bonferroni post hoc analyses showed that both intoxicated and sober cannabis users had higher acceptance rates of new unrelated items compared with the control group (cannabis intoxication: *p* = 0.0002, Cohen’s *d* = 0.83, cannabis sober: *p* = 0.018, Cohen’s *d* = 0.59). However, the two cannabis groups did not differ from one another (*p* = 0.56, Cohen’s *d* = 0.23).

### Net accuracy

We conducted a one-way ANOVA on net accuracy and detected a statistically significant difference between the three groups, with a medium effect size (*F*(2,153) = 4.79, *p* = 0.01, partial eta squared = 0.06). The Bonferroni post hoc analyses showed that the cannabis intoxication group was less accurate compared with the control group (*p* = 0.01, Cohen’s *d* = 0.63), but no difference was found for the cannabis sober group compared with the control (*p* = 0.076, Cohen’s *d* = 0.44) or the cannabis intoxication group (*p* = 0.99, Cohen’s *d* = 0.11).

### Exploratory analyses

Since no statistically significant differences were detected between the experimental and coffee shop control groups in terms of false memory propensity for critical lures, exploratory analyses were conducted to examine factors that might have contributed to this outcome. The two groups of cannabis users, both intoxicated and sober, were collapsed into groups of light (*n* = 30) and heavy (*n* = 73) cannabis users, according to their response to the question whether they consider themselves to be a light or heavy user. To ensure that this division was warranted, these groups’ consumption patterns were compared: light users indicated consuming cannabis on average 5.0 times and consuming on average 3.4 g per week compared with heavy users who reported using cannabis 6.2 times and an average quantity of 7.9 g per week (*t* (36.12) = − 2.65, *p =* 0.012, Cohen’s *d* = 0.73; and *t* (100.27) = − 4.48, *p* < 0.001, Cohen’s *d* = 0.70, respectively).

DRM true and false memory performance was then compared between light and heavy users using independent samples *t* tests. A statistically significant difference was detected between the two groups only for the measure of net accuracy (*t* (101) = − 2.51, *p* = 0.01, Cohen’s *d* = 0.97). An inspection of means revealed that light users demonstrated lower accuracy (*M* = 0.62; *SD* = 0.09; 95% CI = 0.58, 0.65) compared with the heavy users (*M* = 0.66; *SD* = 0.08; 95% CI = 0.64, 0.68). No statistically significant differences were detected with regard to the other DRM measures.

Moreover, as reported above, a multivariate GLM analysis with all four DRM parameters as DVs, group as a fixed factor, and sex as a covariate was conducted. It was inspected whether having sex as a covariate in the model would change any of the between-groups effects. With sex as a covariate in the model, no statistically significant difference of group on net accuracy was detected anymore (*p* = 0.18). All other effects remained unchanged.

## Discussion

The present field study was designed to assess whether cannabis use increases the susceptibility to false memory formation. To allow differentiation of acute and residual effects of cannabis, we compared true and false recognition memory performance in intoxicated and sober regular consumers of cannabis with non-user controls on the DRM paradigm. Contrary to expectations, cannabis users did not demonstrate an increase in false memory rates for critical lures, relative to controls. However, both intoxicated and sober cannabis users showed elevated false alarm rates in response to new, unrelated items. Moreover, no group differences were detected with regard to true memory performance (hit rates), but the control group demonstrated higher net accuracy in memory performance compared with intoxicated cannabis users.

We found no evidence that cannabis use increases recognition of critical lures. At first instance, this finding seems to conflict with the findings by Riba et al. (2015) who reported elevated susceptibility to false memories in abstinent cannabis consumers. However, a closer inspection of the differences in methodology between the two studies may provide an explanation. In the study by Riba et al., a modified version of the DRM paradigm was used, exposing participants to 20 word lists each containing 4 associated words, which were preceded by an announcement of the list name (e.g., farm animals: horse, hen, sheep, goat; see supplementary materials of Riba et al. 2015). The recognition test then included lure items, which were words categorically related to the presented items (e.g., cow, pig), in addition to old, presented words and new, unrelated items. In contrast, the classic version of the DRM paradigm was employed in our study, where participants were presented with lists of 10 words that all primed identical critical lures, and the lists consisting of the first associates of the critical lure based on association word norms (Stadler et al. 1999). As such, the study by Riba et al. (2015) therefore did not include standard critical lures under the definition of the classical DRM paradigm, but words that were related to previously learned words but were not previously primed in the study phase.

DRM lists and the so-called category lists such as the ones used by Riba et al. thus differ in that DRM lists typically contain multiple different associative relations (synonyms, antonyms, concept relations, etc.) whereas category lists are restricted to only one level of association, that is taxonomy (Knott et al. 2012). Moreover, DRM lists are typically higher in backward associative strength (BAS), a measure of strength of the associative connections from study words to the critical lures. Research has shown that higher BAS leads to higher rates of false memories (Knott et al. 2012; Roediger et al. 2001). Although impossible to compare directly as numbers for BAS are missing in Riba et al.’s study, it can be argued that the two measures of false memory differ in associative strength, as the lures used in Riba et al.’s study can be considered less strongly associatively related to the initially presented lists, compared with the critical lures used in our study. The premise that high BAS tends to elicit high rates of false memories is also mirrored in the fact that in our study, false memory rates were rather high in all groups (65–69%), whereas they were relatively low in the Riba et al. study (20–30%).

On the other hand, a medium-to-large effect was found in cannabis use on false recognition of non-presented, associatively unrelated items, with cannabis users showing elevated false alarm rates. This effect fits well with previous findings reported in the literature of studies using non-DRM word list tasks, where acute cannabis intoxication was found to induce elevated intrusions and false alarms of new, unrelated stimuli (e.g., Hart et al. 2010). True recognition performance in our study was unimpaired by cannabis use, which mirrors other studies such as Riba et al. (2015). As mentioned in the introduction, recognition memory has only inconsistently been found to be impaired by cannabis use, a finding that has been reported both in acute and in long-term studies (Broyd et al. 2016; Solowij and Battisti 2008). However, it was found that net accuracy was highest in the control group, and although pairwise comparisons only detected a statistically significant difference between controls and intoxicated cannabis users, this indicates that cannabis impairs overall recognition accuracy.

Two theoretical frameworks can be used to explain the formation of false memories as elicited by the DRM paradigm: associative-activation theory (AAT; Howe et al. 2009) and fuzzy-trace theory (FTT; Brainerd et al. 2008). According to AAT, processing one word activates a corresponding node (i.e., concept) and spreads activation to surrounding, interconnected nodes within one’s semantic network (i.e., knowledge base). False memories can be produced if spreading activation has automatically activated neighboring but non-presented information, leading to false memories. According to FTT, events are encoded into two types of memory traces: verbatim and gist. The verbatim trace contains item-specific details of an event, while the gist trace captures the underlying meaning of the stimulus. Because verbatim traces fade quickly over time, people rely on gist traces when retrieving memories, thereby enhancing false memory formation (Brainerd and Reyna 2002).

Given that critical lure recognition did not differ in the cannabis use groups relative to controls, it does not appear that cannabis use enhanced activation for the related lure words in memory or produced an over-reliance on gist memory traces. However, given the observed increase in unrelated word recognition and the decrease in net accuracy for the cannabis use groups, it may be the case that cannabis impairs processing of the word lists during encoding (i.e., insufficient processing prevents either extensive activation or strong verbatim and gist trace formation). The finding that light cannabis users had worse net accuracy than the heavy users may also support this suggestion as light users are arguably less tolerant to the effects of THC than the heavy users (Ramaekers et al. 2009), so any cannabis effects would be more pronounced in the light use group, i.e., decreased processing in the light use group.

According to AAT, false memories or false alarms depend on the strength of association. DRM lists with high BAS may result in a stronger spreading activation, leading both controls and cannabis users alike to be certain that they remember the critical lure. They might receive a feeling of familiarity when being exposed to the critical lure. On the other hand, if there is no association as in the present study, or a lesser degree of association as in Riba et al.’s study, the level of uncertainty is greater, and thus individuals who are acutely intoxicated or have residual levels may exhibit a tendency toward more liberal responding. Multiple explanations can be put forward for this liberal responding. According to Ranganathan and D’Souza (2006), cannabinoids may induce increased intrusions due to increased mental activity, leading to irrelevant associations. In line with this idea, a recent animal study with cannabinoid receptor type 1 (CB1) knockout mice showed that hippocampal CB1 receptor activation increases the formation of incidental associations (Busquets-Garcia et al. 2018). Alternatively, cannabis use has been associated with increased impulsivity in decision-making (e.g., Metrik et al. 2012; Ramaekers et al. 2009). When making decisions under conditions of uncertainty, this may play out as a lowered decision threshold resulting in greater liberal acceptance of new information.

The current study has several strengths and novelties. Previous studies have been useful in illuminating the role of cannabis in false memory production but have several drawbacks: Specifically, in Riba et al. (2015), the participants were abstinent for at least 4 weeks, making it difficult to determine acute intoxication effects, whereas in Ballard et al. (2012), the participants received a specific dose that may not account for individual differences in tolerance levels. While the Vredeveldt et al. (2018) study advanced on this design by testing participants who chose their own cannabis dose, false memory was not measured directly using a method known to successfully lead to reliable levels of false memories. These drawbacks have been addressed in the current design. In a between-subjects design, we compared groups of acutely intoxicated individuals who use cannabis regularly, sober individuals who use regularly, and individuals without a history of cannabis use. This allowed the distinction between acute and residual effects of cannabis use. Moreover, the study was conducted in a naturalistic setting, maximizing ecological validity, as in a coffee shop, people are more likely to consume a dose specific to their tolerance levels.

However, the study is not without limitations. There was an unequal distribution of sex across groups, with the two cannabis groups consisting largely of male participants, while the control group had a higher proportion of female participants. Although this mirrors findings of cannabis use being more prevalent in males (e.g., Cuttler et al. 2016), it might pose a confounding factor, especially since when sex was included as a covariate in the analyses, no statistically significant group difference was detected for net accuracy anymore. It should be noted though that previous research gives no reason to expect sex effects in DRM performance (Bauste and Ferraro 2004; Seamon et al. 2002). A study by Dewhurst et al. (2012) found a sex difference but only with regard to negative stimuli while there was no difference for neutral lists. Nevertheless, future studies may need to account for sex differences by recruiting a more balanced sample.

Furthermore, as encoding and retrieval occurred in the same session (approximately 10 min apart), it is not clear whether cannabis impacts the encoding or retrieval of experiences. The previously described studies by Ballard et al. (2012) and Doss et al. (2018) separately examined the effects of THC on encoding and recognition testing 2 days apart, meaning encoding occurred during intoxication and retrieval while sober, and vice versa. Doss et al. found that THC at retrieval increased false memory effects whereas Ballard et al., if anything, found reduced false memory effects of THC during encoding. Future studies could investigate the issue of different memory stages and cannabis further by separating the encoding and testing phases with a longer time interval and varying the timing of intoxication. Future studies should also include an additional word category consisting of related but not critical lures, similar as in the study by Riba et al. (2015), to see whether the results converge. Measures of recall rather than just recognition memory, and metacognitive measures such as assessments of confidence would allow for a more comprehensive understanding of the effects of cannabis on multiple memory processes.

Finally, this study has important implications for legal, forensic as well as clinical settings. If cannabis users, intoxicated or sober, have a greater tendency for liberal responding when uncertain, this may have consequences in such settings. When presented with new, irrelevant information, they might be more likely to accept this new information as true or familiar, resulting in erroneous reporting. Even though DRM false memory seems far removed from autobiographical memory for a prolonged event such as a crime, the paradigm preserves an essential property of everyday false memories, namely that they arise from meaning relations (Brainerd 2013). Spontaneous false memories such as those in the DRM can arise in and have been relevant to legal cases (Brackmann et al. 2016; Howe et al. 2017; Otgaar et al. 2019), underlining the importance of the current study.

## Appendix

**Table 3:** Study phase DRM lists

List/critical lure	Word	BAS
Bread	Butter	0.364
	Food	0.000
	Eat	0.000
	Sandwich	0.067
	Flour	0.142
	Dough	0.31
	Crust	0.243
	Slice	0.048
	Loaf	0.552
	Toast	0.364
	Mean	0.209
Cold	Hot	0.676
	Snow	0.199
	Winter	0.277
	Ice	0.364
	Wet	0.108
	Weather	0.032
	Freeze	0.461
	Air	0.000
	Arctic	0.642
	Frost	0.37
	Mean	0.313
Doctor	Nurse	0.574
	Sick	0.031
	Medicine	0.152
	Hospital	0.027
	Ill	0.000
	Patient	0.365
	Office	0.014
	Surgeon	0.479
	Clinic	0.3
	Cure	0.028
	Mean	0.197
Fruit	Apple	0.154
	Orange	0.194
	Kiwi	0.709
	Citrus	0.426
	Ripe	0.151
	Pear	0.347
	Banana	0.215
	Berry	0.298
	Cherry	0.168
	Salad	0.000
	Mean	0.266
Man	Husband	0.018
	Uncle	0.07
	Male	0.131
	Father	0.048
	Strong	0.02
	Friend	0.000
	Beard	0.055
	Person	0.122
	Muscle	0.048
	Suit	0.074
	Mean	0.059
Girl	Dolls	0.199
	Female	0.098
	Young	0.000
	Dress	0.063
	Pretty	0.149
	Hair	0.000
	Beautiful	0.049
	Cute	0.035
	Daughter	0.042
	Sister	0.041
	Mean	0.068
Sleep	Bed	0.638
	Rest	0.475
	Awake	0.618
	Tired	0.493
	Dream	0.247
	Blanket	0.024
	Snore	0.439
	Nap	0.73
	Peace	0.000
	Yawn	0.235
	Mean	0.390
Soft	Light	0.000
	Pillow	0.236
	Plush	0.178
	Cotton	0.166
	Fur	0.061
	Touch	0.061
	Fluffy	0.266
	Feather	0.045
	Furry	0.061
	Downey	0.221
	Mean	0.130
Sweet	Candy	0.336
	Sugar	0.433
	Taste	0.071
	Tooth	0.000
	Honey	0.451
	Chocolate	0.101
	Heart	0.000
	Cake	0.027
	Tart	0.223
	Pie	0.000
	Mean	0.164
Thief	Steal	0.089
	Robber	0.361
	Burglar	0.257
	Money	0.000
	Bad	0.000
	Rob	0.074
	Jail	0.013
	Gun	0.000
	Crime	0.028
	Criminal	0.051
	Mean	0.087

All BAS values are drawn from Roediger et al. (2001)

**Table 4:** Recognition phase DRM lists

Recognition phase					
1)	Strong^a^	21)	Bad^a^	41)	Tiger^b^
2)	Butter^a^	22)	Garbage^b^	42)	Dream^a^
3)	Table^b^	23)	Circus^b^	43)	Plush^a^
4)	Crown^b^	24)	Shoe^b^	44)	Taste^a^
5)	Bread^c^	25)	Apple^a^	45)	Honey^a^
6)	Eat^a^	26)	Medicine^a^	46)	Sweet^c^
7)	Nose^b^	27)	Fur^a^	47)	Steal^a^
8)	Flour^a^	28)	Fruit^c^	48)	Train^b^
9)	Hot^a^	29)	Waste^b^	49)	Shirt^b^
10)	Candy^a^	30)	Captain^b^	50)	Sleep^c^
11)	Doctor^c^	31)	Ill^a^	51)	Dolls^a^
12)	Flexible^b^	32)	Ink^b^	52)	Water^b^
13)	Winter^a^	33)	Ripe^a^	53)	Hand^b^
14)	Young^a^	34)	Wine^b^	54)	Pollution^b^
15)	Stars^b^	35)	Cold^c^	55)	Nurse^a^
16)	Girl^c^	36)	Husband^a^	56)	Light^a^
17)	Man^c^	37)	Pretty^a^	57)	Smoke^b^
18)	Wet^a^	38)	Bed^a^	58)	Burglar^a^
19)	Awake^a^	39)	Eye^b^	59)	Kiwi^a^
20)	Soft^c^	40)	Male^a^	60)	Thief^c^

Stimuli were presented in the listed order

^a^Previously presented (old) word

^b^New, unrelated word

^c^Critical lure
